# Doubly Blinded: An Uncommon Cause of Acute Visual Loss Due to Orbital Compartment Syndrome

**DOI:** 10.3390/clinpract11020046

**Published:** 2021-05-24

**Authors:** Khalid Sawalha, Devi Nair

**Affiliations:** 1 Internal Medicine Division, White River Health System, Batesville, AR 72501, USA; 2 Electrophysiology Division, White River Health System, Batesville, AR 72501, USA; DGNair1@yahoo.com

**Keywords:** retrobulbar hematoma, orbital compartment syndrome

## Abstract

A 68-year-old female patient with a past medical history of atrial fibrillation on anticoagulation regimen with Apixaban and Clopidogrel presented for her scheduled Watchman device implantation. The device was indicated as patient was high risk for falling. Successful implantation of the left atrial appendage device was carried out, and the patient was sent to the floor. One hour after the procedure, the patient started having left-sided diplopia along with severe eye pain. An immediate CT scan of the head showed left superior orbital mass, concerning for hematoma. Urgent left canthotomy with cantholysis was conducted bedside. However, despite early interventions, the patient’s vision was lost.

## 1. Introduction

Retrobulbar hemorrhage (RBH) is a very rare complication with an incidence reported to be lower than 1% of cases related to blunt facial trauma [1]. It is a rapidly progressing, sight-threatening emergency that results in an accumulation of blood in the retrobulbar space. This blood accumulation acts as a compartment syndrome in which increased intra-ocular pressure (IOP) may result in damage to intra-orbital structures such as the optic nerve. This, in turn, can result in irreversible damage if not managed quickly [2]. Early recognition of symptoms and clinical signs is the mainstay of management. However, despite the urgent interventions, complications might be permanent, unfortunately.

## 2. Case Presentation

**Figure 1:**
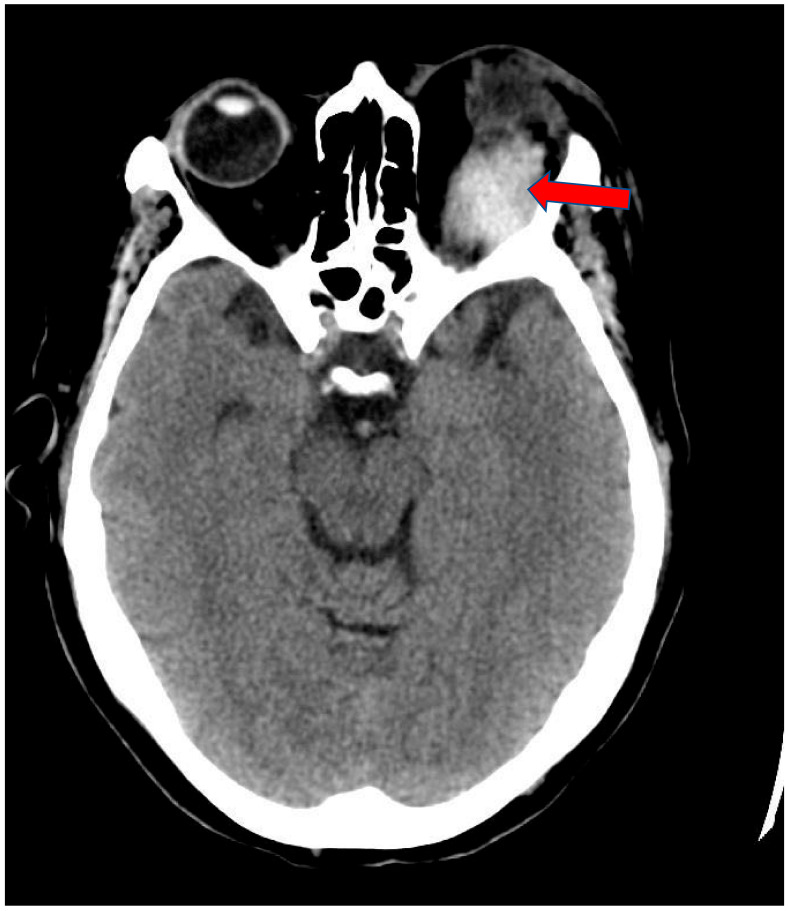
Axial view of Brain CT scan showing left periorbital soft tissue swelling with hyper dense mass. This appears to be located above the superior rectus muscle (Arrow).

**Figure 2:**
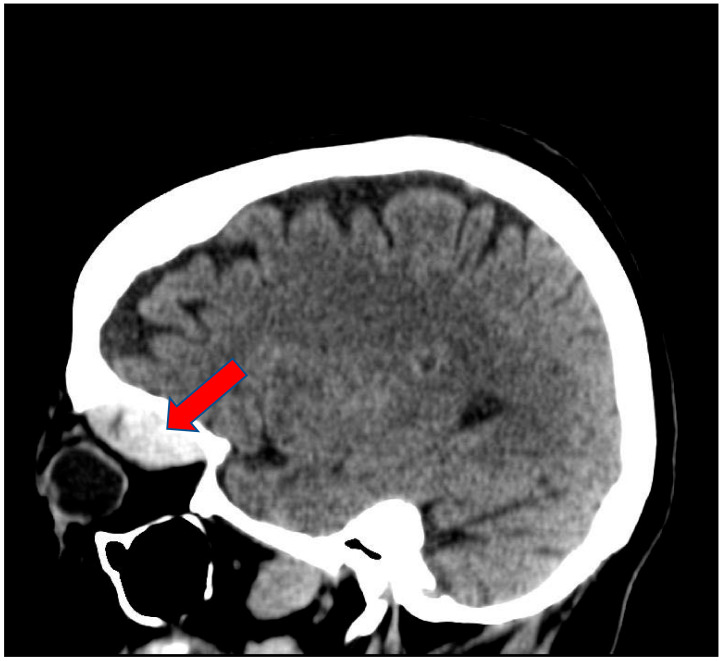
Sagittal view of Brain CT scan showing left soft tissue swelling with hyper dense mass in the left superior orbit measuring 3.7 × 2.9 × 1.5 cm (Arrow).

A 68-year-old white female patient with a past medical history of atrial fibrillation, hypertension and anxiety presented for her scheduled Watchman device implantation. Her risk of bleeding secondary to falling was high given her multiple recent falls at home. Four days prior to her procedure, she recalled having a left-sided headache for which she did not seek medical attention as she related it to being stressed about the procedure. Her anticoagulation regimen included Apixaban and Clopidogrel, of which both were held five days prior to the procedure. The patient underwent successful placement of the left atrial appendage device with no immediate complications. During the procedure she received 10,000 units of Heparin and with an activated clotting time of 312 s (150–600 s). One hour after the procedure, she started complaining of nausea and left-sided diplopia along with left eye swelling and severe pain. Physical examination revealed left eye edema, peri-orbital ecchymosis, proptosis, and loss of pupillary response. Left ophthalmic examination revealed corneal edema and cupping of the optic disc. Left ocular movement was limited to all direction. The right eye was normal. An immediate CT scan of the head revealed left periorbital soft tissue swelling with a left superior orbital hyperdense mass, concerning for hematoma, measuring 3.7 × 2.9 × 1.5 cm (Figure 1 and Figure 2). Urgent left canthotomy with cantholysis was conducted bedside, in which both intra-ocular pressure and pain were significantly reduced from 52 mm hg to 24 mm hg (10–21 mm hg). Later, despite early interventions, the patient’s left vision was lost completely.

## 3. Discussion

Spontaneous retrobulbar hemorrhage is a rare sight-threatening phenomenon that is most commonly associated with orbital trauma, repair of orbital floor fractures, ocular surgery, endoscopic sinus surgery, retrobulbar injections, and other head and neck procedures, including blepharoplasty, where bleeding may occur in proximity to the globe. Rarely, it occurs in susceptible individuals after general anesthesia as a result of coughing, straining, vomiting, or extremely elevated blood pressure, particularly in the presence of anticoagulants such as Aspirin and Coumadin. Incidence varies among surgical procedures, occurring in 0.04 percent of blepharoplasties [3], 0.43 percent of endoscopic sinus surgeries [4], and in up to 3.2 percent of orbital floor fractures [5].

Onset of symptoms typically occurs within three hours of surgery and the vast majority within 24 h; however, onset may occur as many as nine days after surgery [6,7]. Clinicians should have a high index of suspicion for this complication after head and neck procedures, especially after orbital floor repair [8,9,10,11,12].

The clinical presentation may be dramatic in appearance, and the diagnosis is made based on clinical signs and symptoms. Proptosis with severe stabbing pain, vision loss, and a sensation of pressure are common. Patients may also experience nausea and vomiting, diplopia, and visual flashes [13]. External examination of the eye may reveal eyelid hematoma or ecchymosis, subconjunctival hemorrhage, proptosis, or ophthalmoplegia. If the optic nerve is compromised, pupillary light reflexes will be abnormal, with a relative afferent pupillary defect. Brain imaging is necessary to assess orbital structures as well as presence of pathological lesions. CT or MRI can confirm the diagnosis and exclude other potential causes [6]. Due to the emergent nature of retrobulbar hemorrhage in cases associated with trauma, CT is preferred because it shows the bony anatomy in a timely manner. In cases where vascular anomalies are suspected, MRI is the choice. Emergency ophthalmology consultation is indicated if the diagnosis is suspected.

RBH occurs in a confined orbital space that is surrounded by a bony wall; this results in a rapid elevation of intra-orbital pressure. This pressure is transmitted to the eye and optic nerve causing ischemia through orbital compartment syndrome (OCS). OCS is a true ophthalmologic emergency that requires immediate decompression with a lateral canthotomy and inferior cantholysis, and may be performed at the bedside with local anesthesia. Emergency surgical decompression should not be delayed for imaging or administration of topical or systemic medications aimed at lowering intraocular pressure. Evacuation of the hematoma may be performed subsequent to decompression. Prognosis for vision is dependent on the time from the onset of symptoms to decompression, with poor outcomes reported in symptom-to-treatment intervals as short as four hours. Retrobulbar hematoma is responsible for almost half of all cases of visual loss associated with repair of orbital floor fractures [5].

Prevention includes primarily surgical hemostasis, avoidance of excessive coughing and straining with emergence, and retching in the recovery room [8,9,10,11,12]. In our case, despite early recognition of symptoms and prompt surgical intervention within the reported literature time frame, the patient still endured loss of vision. Therefore, patients with a potential risk and a high suspicion of orbital compartment syndrome, such as those on blood thinners or undergoing procedures in which they will receive anticoagulants, should be systematically monitored for signs and symptoms, and assessed closely.

## 4. Conclusions

This case sheds light on the urgent nature of orbital compartment syndrome and the importance of early recognition and intervention. Teaching, training, and early surgical decompression is reported to be the only solution to save the blind eye, but despite this, it may remain blind.

## Data Availability

Data available upon request.
